# Impact of adipose tissue or umbilical cord derived mesenchymal stem cells on the immunogenicity of human cord blood derived endothelial progenitor cells

**DOI:** 10.1371/journal.pone.0178624

**Published:** 2017-05-31

**Authors:** Kefang Tan, Ke Zheng, Daiye Li, Haiyuan Lu, Siqi Wang, Xuan Sun

**Affiliations:** 1 Institute of Reproductive and Stem Cell Engineering, School of Basic Medical Sciences, Central South University, Changsha, Hunan, China; 2 National Engineering and Research Center of Human Stem Cell, Changsha, Hunan, China; 3 Xiangya Hospital, Central South University, Changsha, Hunan, China; Universita degli Studi di Torino, ITALY

## Abstract

The application of autologous endothelial progenitor cell (EPC) transplantation is a promising approach in therapeutic cardiovascular diseases and ischemic diseases. In this study, we compared the immunogenicity of EPCs, adipose tissue (AD)-derived mesenchymal stem cells (MSCs) and umbilical cord (UC)-derived MSCs by flow cytometry and the mixed lymphocyte reaction. The impact of AD-MSCs and UC-MSCs on the immunogenicity of EPCs was analyzed by the mixed lymphocyte reaction and cytokine secretion *in vitro* and was further tested by allogenic peripheral blood mononuclear cell (PBMC) induced immuno-rejection on a cell/matrigel graft in an SCID mouse model. EPCs and AD-MSCs express higher levels of MHC class I than UC-MSCs. All three kinds of cells are negative for MHC class II. UC-MSCs also express lower levels of IFN-γ receptor mRNA when compared with EPCs and AD-MSCs. EPCs can stimulate higher rates of proliferation of lymphocytes than AD-MSCs and UC-MSCs. Furthermore, AD-MSCs and UC-MSCs can modulate immune response and inhibit lymphocyte proliferation induced by EPCs, mainly through inhibition of the proliferation of CD8+ T cells. Compared with UC-MSCs, AD-MSCs can significantly improve vessel formation and maintain the integrity of neovascular structure in an EPC+MSC/matrigel graft in SCID mice, especially under allo-PBMC induced immuno-rejection. In conclusion, our study shows that AD-MSC is a powerful candidate to minimize immunological rejection and improve vessel formation in EPC transplantation treatment.

## Introduction

Endothelial progenitor cells (EPCs) are considered a cellular resource for differentiation into vascular endothelial cells [1]. EPCs can promote neovascularization at the site of vascular injury or *de novo* neovascularization [2]. Many studies suggested that transplanted EPCs could regenerate damaged vessels and ameliorate symptoms of ischemic diseases [3]. Pre-clinical studies indicated that implantation of EPCs could improve vascularization, thus improving the quality of life for patients who suffer from peripheral arterial diseases [4]. These studies showed transplantation of autologous EPCs could become a new cell-based therapeutic strategy for vascular disease or ischemic disease treatment. However, in most cases, EPCs derived from these patients were dysfunctional or hard to proliferate [5]. Thus transplantation of allogenic EPCs may provide a novel and useful potential therapeutic technique for treating vascular diseases or ischemic diseases.

It is well known that allografts can lead to immunological rejection and greatly reduce therapeutic efficiency [6], which is another major obstacle in the clinical application of allo-EPCs. Cord blood-derived EPCs are the most easily obtainable and the most commonly used allogenic EPC. However, the immunogenicity of human cord blood derived EPCs is not fully elucidated. Most related studies have focused on the neovascularization function of EPCs or auto-transplantation of peripheral blood- or bone marrow-derived EPCs [7,8]. However, these kinds of EPCs are not sufficient for auto-transplantation even after amplification *in vitro*. Furthermore, the function of EPCs in older patients with cardiovascular disease or ischemic disease is significantly impaired [9]. In many cases, allogenic EPCs are necessary to treat vascular diseases.

Mesenchymal stem cells (MSCs) have self-renewal abilities and can differentiate into many different cell types. MSCs express low levels of MHC class I and are negative for MHC class II, CD80, and CD86. Therefore, MSCs are considered to be immune privileged. MSCs are able to modulate the immune reaction *in vitro* and *in vivo* through cell-cell contact and secretion of soluble cytokines [10,11]. MSCs are used as promising candidate cells for preventing rejection in organ transplantation and the treatment of autoimmune disease [12,13].

In this study, we compared the immunogenicity of human umbilical cord blood-derived EPCs, human adipose-derived MSCs (AD-MSCs) and human umbilical cord-derived MSCs (UC-MSCs). Furthermore, we detected the immune-modulatory effects of AD-MSCs and UC-MSCs on EPCs *in vitro* and *in vivo*.

## Materials and methods

### Mouse experiments

Six-week-old male SCID/beige immunoincompetent mice were purchased from Charles River Laboratories and housed under specific pathogen-free conditions with a 12-hour dark/light cycle and free access to food and water, and cared for according to Guide for the Care and Use of Laboratory Animals, 8^th^ edition, as published by the National Academies Press(US). The mice were used between 6–7 weeks after birth and weighed 18–21 g. All animal procedures were approved by the Committee on the Ethics of Animal Experiments of the Central South University.

### Cell culture

Ethics approval and oversight about human tissues was obtained from the Reproductive and Stem Cell Engineering Ethics Committee of Central South University and the Reproductive and Genetic Hospital of China International Trust and Investment Corp.-Xiangya.

EPCs were isolated and cultured as previously described [14]. Briefly, human umbilical cord blood was collected from full-term pregnancies with informed consent. Blood was diluted with DPBS, overlaid with an equal volume of Ficoll (China) and centrifuged for 15 min at room temperature. Mononuclear cells (MNCs) were isolated and washed twice with DPBS, then cultured on 0.1% fibronectin pre-coated plates in endothelial growth medium -2 (EGM-2, Lonza, USA). Non-adherent cells were discarded after 48 h. Culture medium was changed every other day. Cultures were maintained until the outgrowth of cobblestone-like colonies. Cells were grown to 90% confluences and digested with TrypLE (Invitrogen, USA) and passaged.

Human abdomen adipose tissue was collected with informed consent from parturients who underwent caesarean section. The adipose tissue was carefully cut into 1 mm^3^ pieces and digested by 0.1% collagenase type I (Sigma, USA) for 4 h. Cell suspensions were collected and centrifuged, and the cells were washed twice with DPBS, then cultured in DF/12 (Gibco, USA) supplemented with 10% FBS (Gibco, USA) and 10 ng/ml bFGF (Invitrogen, USA). Confluent cells were digested with Trypsin/EDTA (Gibco, USA) and passaged.

UC-MSCs were isolated and cultured as previously described [15]. Human umbilical cord tissue was collected from full-term pregnant women who gave informed consent. Umbilical cord veins and arteries were stripped, tissues were cut into 2 cm pieces and enzymatically digested for 16 h at 37°C. Cell suspensions were collected and centrifuged, and the cells were washed twice, then cultured in DF/12 supplemented with 10% FBS and 10 ng/ml bFGF. Adherent cells were cultured and expanded.

In some experiments, cultured EPCs, AD-MSCs or UC-MSCs were pretreated with INF-γ (10 ng/ml, Gibco, USA) for 48 h before harvest.

### Flow cytometry

Single-cell suspensions were generated by digestion of confluent cells. A total of 10^6^ cells were incubated with antibodies or an isotype control antibody at 4°C for 30 min in the dark. Samples were washed twice with PBS and analyzed with an FACS Calibur flow cytometer (Becton Dickinson). Specific antibodies used in these analyses were CD34-PE, CD29-PE, CD90-PE, CD45-FITC, CD73-FITC, CD105-FITC, CD14-PE, CD19-PE, CD4-PE, CD8-APC (Biolegend, USA), VEGFR-2-PE, CD144-PE (BD, USA), vWF-FITC (Abcam, USA), CD31-FITC (eBioscience, USA), MHC I-PE, MHC II-PE, CD40-PE, CD80-PE, CD86-PE (Miltenyi Biotec) and isotype control IgG-PE (from Miltenyi Biotec or Biolegend, USA), IgG-FITC (from ebioscience or Biolegend, USA), Flow cytometric data were analyzed using BD CELLQuest software.

### Functional analysis for EPCs and MSCs

Confluent EPCs (passage 2–4) were washed with DPBS, fixed with 4% paraformaldehyde (Sigma, USA), then incubated with Ulex Europaeus agglutinin 1(UEA-1, Vector Laboratories, USA) in the dark at 37°C for 20 min. Cells were washed twice with DPBS, fixed with 4% paraformaldehyde for 30 min, DAPI staining (Sigma, USA) was used to mark nuclei. Samples were observed under fluorescence microscopy.

Confluent EPCs (passage 2–4) were incubated with Alexa Flour 488 acetylated low-density lipoprotein (DiI-ac-LDL; Molecular Probes/Life Technologies) at 37°C for 4 h. Cells were washed twice with DPBS and fixed with 4% paraformaldehyde for 30 min; DAPI staining was used to mark nuclei. Samples were observed under fluorescence microscopy.

MSCs were induced to differentiate into adipose cells or osteocytes as previously described [15]. Briefly, the MSCs were induced in adipogenic differentiation medium or osteogenic differentiation medium for 14 days. Induced adipose cells were detected by oil red O staining (Sigma, USA) at 0, 7 and 14 days. Induced osteocytes were detected at 0, 7 and 14 days using a BCIP/NBT kit (Invitrogen, USA) to test for the expression of alkaline phosphatase.

### Quantitative real-time polymerase chain reaction (qRT-PCR)

Total RNA was extracted from cultured cells using the TRIzol reagent system (Invitrogen, USA). A total of 1.0 μg total RNA was reverse-transcribed into cDNA with the Reverse Transcription system (Promega, USA) following the manufacturer’s instructions. cDNA samples were subjected to PCR amplification with specific primers and the GoTaq qPCR Master Mix (Promega, USA). PCR primers are described in Table 1. Reactions were performed in a Roche Lightcycler 480. All samples were analyzed in triplicate. The value of the threshold cycle of each reaction was compared.

**Table 1:** Primer sets of RT-PCR that used in this experiment.

Gene	Forward primer	Reverse primer	Product size
GAPDH	5’-CTCTGCTCCTCCTGTTCGAC-3’	5’-AAATGAGCCCCAGCCTTCTC-3’	408
IFNGR1	5’-TAAATGGAGACGAGCAGGAAG-3’	5’-TGAATACCAGGCTAAGCACTA-3’	342
IFNGR2	5’-TTTAGAGTCGGGCATTTAAGCA-3’	5’-TCAGGACCAGGAAGAAACAGG-3’	145

### Western blot analysis

Protein samples from culture EPCs or MSCs were extracted using the RIPA lysis buffer (Applygen, Beijing) supplemented with Protease Inhibitor Cocktail Set II (Merck Millipore). Antibodies (all from Proteintech, USA) against interferon gamma receptor 1 from rabbit (IFN-γR1, 1:1000 dilution) and interferon gamma receptor 2 from rabbit (IFN-γR2, 1:1500 dilution), β-actin from mouse (1:4000 dilution), Goat anti-rabbit IgG HRP (1:6000 dilution) were used for testing of protein expression.

### Lymphocyte proliferation assay

EPCs were pre-treated with IFN-γ (10 ng/ml, 48 h) or left untreated. A total of 5×10^4^ cells/well stimulator cells (pre-treated EPCs; un-treated EPCs; MSCs; pre-treated EPCs and MSCs (2:3); un-treated EPCs and MSCs (2:3)) were washed and seeded into a 96-well plate separately. For mixed lymphocyte reactions (MLR), all the attached stimulator cells were pre-treated with 10 μg/ml mitomycin C for 2 h.

Human PBMCs were collected from healthy individuals who gave informed consent. Allo-PBMCs were isolated from human peripheral blood by density gradient centrifugation using Ficoll. Isolated PBMCs were incubated with red blood cell lysis buffer (Qiagen, Germany) for 5 min at room temperature and washed twice with DPBS. A total of 2×10^5^ PBMCs were labeled with CFDA-SE (2 μM, 5 min; Beyotime), added to each well and co-cultured with different stimulator cells. All cells were cultured in RPMI 1640 (HyClone, USA) with 10% FBS and 2 μM L-glutamine (Sigma, USA). PBMCs cultured alone or stimulated by mitogen phytohaemagglutinin (PHA, 5 μg/ml; Sigma, Australia) acted as a negative control and positive control, respectively. After one week of co-culture, suspended PBMCs were harvested; proliferation of lymphocyte and T lymphocyte subsets were analyzed by flow cytometry.

### Vessel formation *in vivo*

SCID/beige immunoincompetent mice were used as transplant recipients for *in vivo* vessel formation. Pentobarbital sodium (60 mg/kg, Sigma, USA) was delivered to each mouse via intraperitoneal injection. The dorsal flank of each mouse was shaved and wiped down with an alcohol pad before implant injection.

Cells (EPCs, EPCs:AD-MSCs (3:2), and EPCs:UC-MSCs (3:2)) were suspended in matrigel (BD, USA) at a final concentration of 1×10^7^ cells/ml according to the manufacturer’s instructions. A total of 200 μl cell suspensions in ice-cold matrigel were injected subcutaneously on the dorsal flank of the mouse, and two grafts were implanted in each mouse. Cell-free matrigel plugs served as controls. After two weeks, the mice were randomly divided into two groups, half of the animals were injected via tail vein with 2×10^6^ PBMCs that were allogeneic to the EPCs and MSCs in 200 μl DPBS. The other half of the animals were injected with an equal volume of DPBS as the control. One week after PBMCs/DPBS injection, the mice were sacrificed by cervical dislocation under deep anesthesia and the grafts were harvested from each flank for histological evaluation.

### Histological evaluation

For histological staining, grafts were fixed in 4% PFA for 1 h and 0.4% PFA overnight. Samples were embedded in paraffin and then sliced into 5-mm sections and stained with hematoxylin and eosin (H&E). For immunofluorescence staining, slides were blocked for one hour with 5% bovine serum albumin (BSA). Rabbit anti-CD31 antibody (from Proteintech, 1:50) and mouse anti-alpha smooth muscle actin (SMAα) antibody (from Abcam, 1:50) were incubated overnight at 4°C according to standard protocols. After washing by PBS for 3 times, secondary antibodies Alexa fluor 594-conjugated goat anti-mouse lgG and Alexa Fluor 488-conjugated Affinipure Goat Anti-Rabbit IgG(H+L) (Proteintech) were incubated for 90 min at 37°C in the dark. After application of DAPI for 10 min, slides were observed under a fluorescence microscope (Motic) based on the tube structure formation and inflammatory infiltration.

### Cytokine production

Supernatants of co-cultured stimulator cells and PBMCs were collected after 6 days and centrifuged for 5 min at 12000 rpm. Cellular debris was discarded. Cytokine production (IL-10, IFN-γ) was determined by MILLIPLEX MAP Human TH17 Magnetic Bead Panel (BD, USA) and analyzed by Luminex according to the manufacturer’s instructions.

### Statistical analysis

The experiments were repeated 3 times. All data were expressed as the means ±SD. For comparisons between two groups, significant differences were measured using Student’s t test. For multiple comparisons, significant differences were measured by one-way ANOVA. A value of p≤0.05 was considered statistically significant.

## Results

### Phenotype and function of EPCs and MSCs

**Fig 1:**
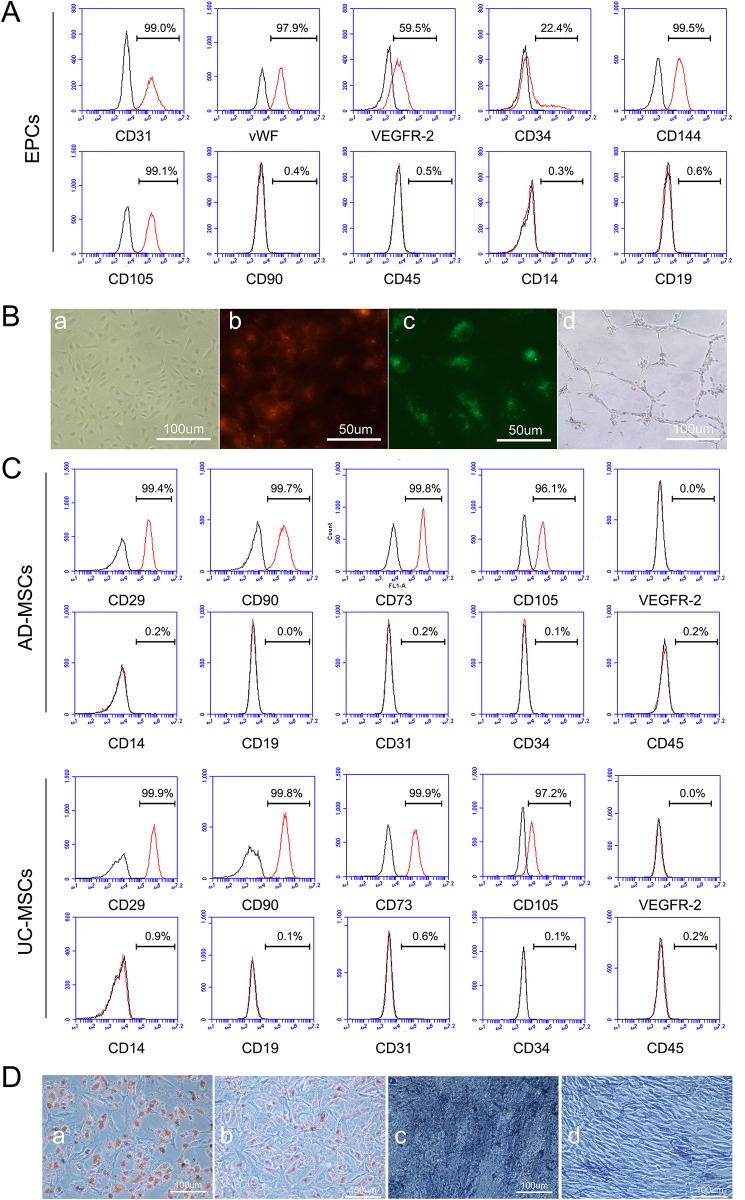
Characterization of EPCs, AD-MSCs and UC-MSCs. A, Expression patterns of endothelial makers on EPCs were analyzed by FACS. EPCs highly expressed CD31, vWF, CD144 and CD105, partly positive expressed VEGFR-2 and CD34, negative for CD90, CD45, CD14 and CD19. B, Biological function of EPCs was identified. a, Representative phase contrast images of cobblestone-like EPCs. b, EPCs bound with UEA-1 (red). c, EPCs incorporated DiI-Ac-LDL (green). d, EPCs formed vascular-like tubes on matrigel. C, Phenotype analysis of AD-MSCs and UC-MSCs by FACS. Both AD-MSCs and UC-MSCs were positive for CD29, CD90, CD73 and CD105, negative for VEGFR-2, CD14, CD31, CD34 and CD45. D, After 14 days of induction, AD-MSCs and UC-MSCs were differentiated into adipocytes and osteocytes. a, Adipogenic induction of AD-MSCs. b, Adipogenic induction of UC-MSCs. c, Osteogenic induction of AD-MSCs. d, Osteogenic induction of UC-MSCs. Adipogenesis was detected by the formation of neutral lipid vacuoles stainable with oil red O (red-orange). Osteogenesis was demonstrated by detection of alkaline phosphatase activity (brown).

EPCs (passage 3) derived from human umbilical cord blood were positive for the endothelial lineage surface markers CD31 (98.7%±0.49), vWF (97.8%±0.26), VEGFR-2 (57.8±1.47), CD34 (25.3%±2.85), CD144(99.4±0.31), and CD105(99.6±0.44), negative for CD90(0.4±0.06), CD45(1.0±0.62), CD14(0.4±0.12) and CD19(1.5±1.14). They could incorporate DiI-Ac-LDL and bind with UEA-1 (Fig 1A and 1B).

AD-MSCs (passage 3) and UC-MSCs (passage 3) were positive for CD29 (99.7±0.27 and 99.9±0.06, respectively), CD90 (99.4±0.29 and 99.8±0.06, respectively), CD73(99.9±0.06 and 99.9±0.06, respectively) and CD105(96.7±0.55 and 97.8±0.55, respectively), but negative for VEGFR-2(0.5±0.55 and 0.1±0.17, respectively), CD14(0.7±0.29 and 1.1±0.47, respectively), CD19(0.6±0.85 and 0.1±0.12, respectively), CD31 (0.7±0.57 and 0.6±0.15, respectively), CD34(0.1±0.06 and 0.1±0.06, respectively) and CD45 (0.6±0.4 and 0.3±0.15, respectively). AD-MSCs and UC-MSCs could be induced to become adipocytes and osteocytes after 7 days differentiation. Adipocyte and osteocyte induction was easier in AD-MSCs than UC-MSCs (Fig 1C and 1D).

### Immunophenotype of EPCs and MSCs

**Fig 2:**
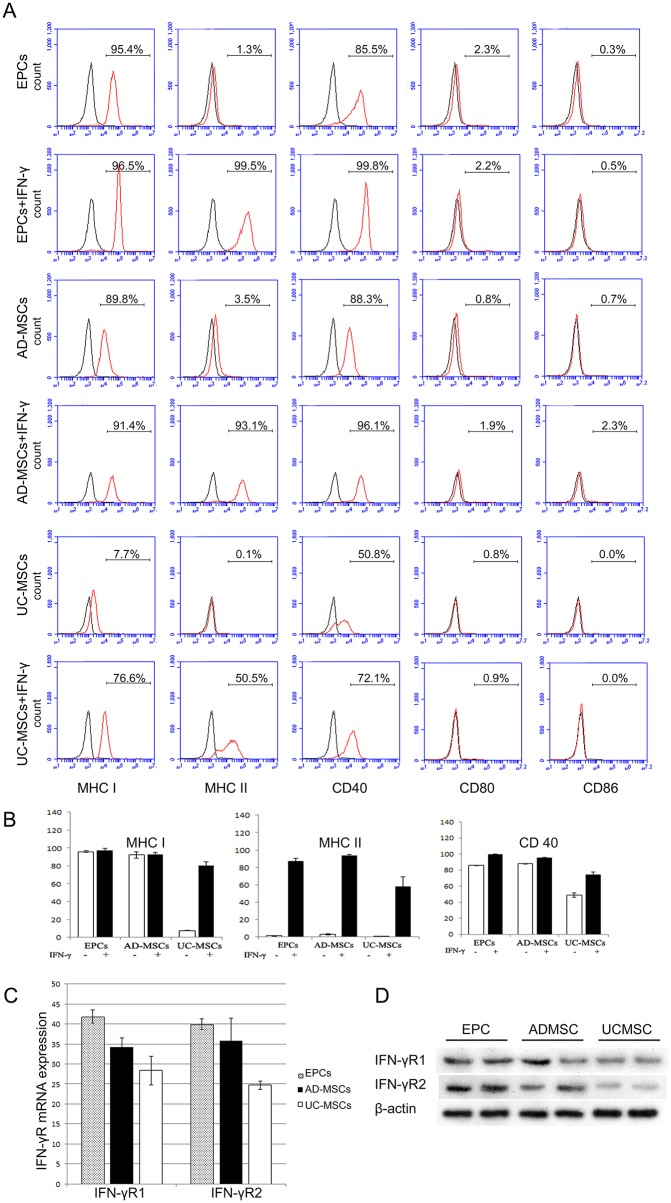
Immuno-phenotyping of EPCs, AD-MSCs and UC-MSCs. A, Typical FACS results of MHC-I, MHC-II, CD40, CD80 and CD86 expression of three kinds of cells, with or without IFN-γ-treatment. B, Statistical analysis of FACS results of MHC-I, MHC-II and CD40 expression of the three kinds of cells, with or without IFN-γ-treatment. EPCs and AD-MSCs expressed significantly higher levels of MHC I and CD40 when compared with UC-MSCs. All three kinds of cells were negative for MHC II, CD80 and CD86. After stimulated with IFN-γ, the expression of MHC I, MHC II and CD40 were up-regulated in all the three kinds of cells, while the induced expression level in UC-MSCs was relatively low compared to AD-MSCs and EPCs (n = 3, P<0.01). C, mRNA levels of IFN-γR1 and IFN-γR2 gene were compared within three kinds of cells. The expression levels of both IFN-γR1 and IFN-γR2 were highest in EPCs, lowest in UC-MSCs (n = 3, P<0.01). D, Protein level of IFN-γR1 and IFN-γR2 were tested by Western blot, and compared within three kinds of cells with Quantity One software. The expression levels of both IFN-γR1 (EPC *vs* UC-MSC, P = 0.03) and IFN-γR2 (EPC *vs* UC-MSC, P = 0.016) were highest in EPCs, lowest in UC-MSCs (n = 3).

Flow cytometric analysis revealed that EPCs and AD-MSCs have similar immunophenotypes. EPCs and AD-MSCs expressed high levels of MHC class I and CD40, while UC-MSCs expressed low levels of MHC I and CD40. EPCs, AD-MSCs and UC-MSCs were all negative for MHC class II, CD80 and CD86. After stimulation by IFN-γ for 48 h, MHC I, MHC II and CD40 were significantly up-regulated in all three kinds of cells, whereas CD80 and CD86 remained unchanged (Fig 2A).

However, compared with IFN-γ-treated EPCs or AD-MSCs, IFN-γ-treated UC-MSCs expressed lower levels of MHC I, MHC II and CD40 (Fig 2B). Furthermore, we investigated IFN-γ receptor mRNA and protein expression of EPCs, AD-MSCs and UC-MSCs. The results indicated that EPCs expressed the highest level of IFN-γ receptors among the three kinds of cell types, while UC-MSCs showed the lowest expression level (Fig 2C and 2D).

### AD-MSCs and UC-MSCs could inhibit the proliferation of allo-PBMCs *in vitro*

IFN-γ-treated or untreated EPCs were co-cultured with allo-PBMCs for 7 days. Flow cytometry analysis demonstrated that EPCs could stimulate allo-PBMC proliferation. EPCs pre-treated with IFN-γ could stimulate PBMC proliferation more significantly than untreated EPCs.

**Fig 3:**
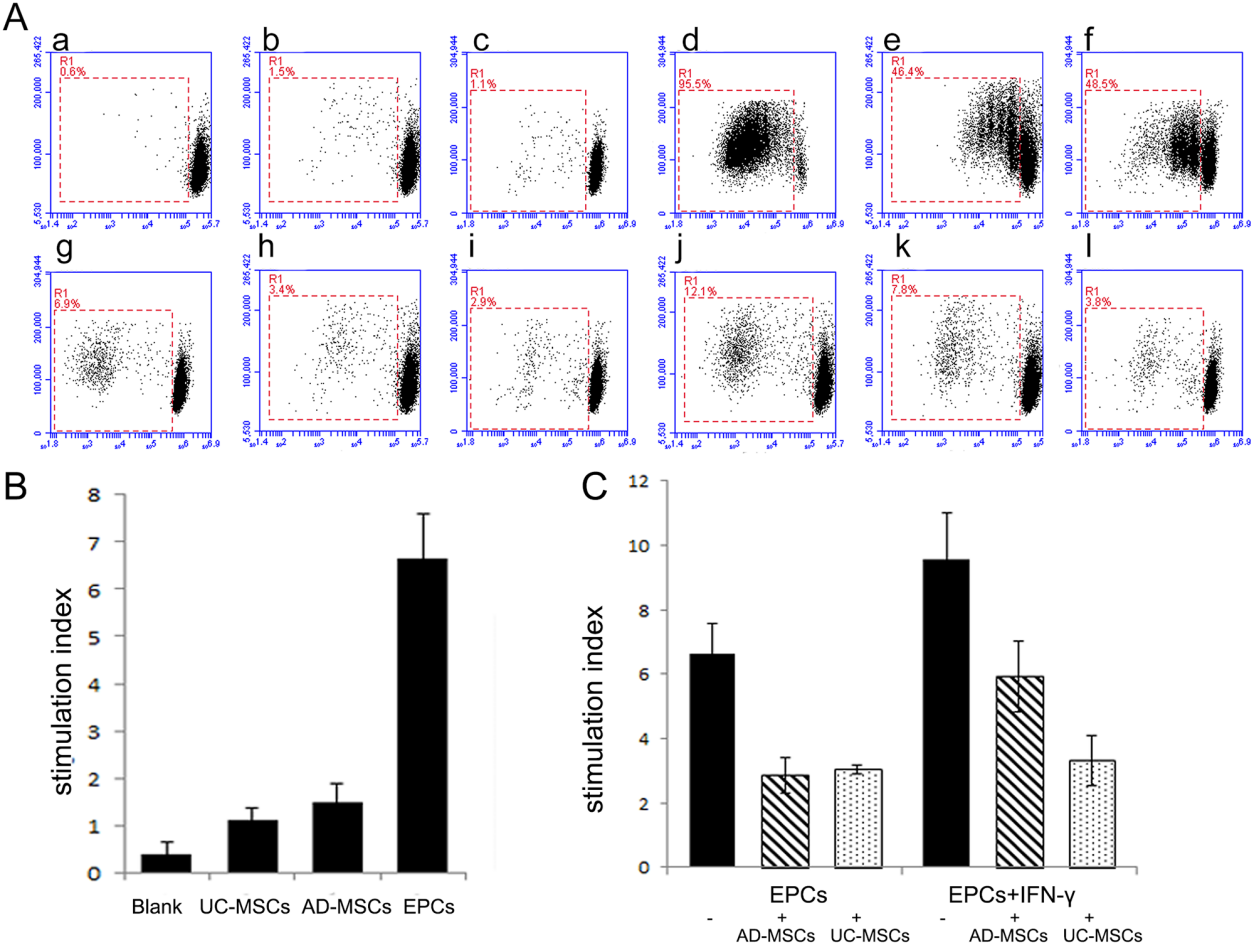
AD-MSCs and UC-MSCs could inhibit the proliferation of allo-PBMCs *in vitro*. Proliferation of allo-PBMCs co-cultured with different stimulating cells was analyzed by CFDA-SE-based proliferation assay. A, Typical results of the mixed lymphocyte reaction (MLR) assay. a-l show allo-PBMC proliferation after co-culture with different stimulators: a, blank as negative control; b, AD-MSCs; c, UC-MSCs; d, PHA as positive control; e, AD-MSCs + PHA; f: UC-MSCs + PHA; g, EPCs; h, EPCs + AD-MSCs; i, EPCs + UC-MSCs; j, IFN-γ-treated EPCs; k, IFN-γ-treated EPCs + AD-MSCs; l, IFN-γ-treated EPCs + UC-MSCs. B and C, The statistical results of MLR assay. B, AD-MSCs and UC-MSCs could slightly stimulate allo-PBMC proliferation. EPCs displayed a significantly stronger stimulating effect than AD-MSCs and UC-MSCs (n = 5, P<0.05). C, IFN-γ-treated EPCs showed a higher stimulating effect than EPCs. AD-MSCs and UC-MSCs could strongly down-regulate the stimulating effect of EPC and IFN-γ-treated EPCs. UC-MSCs have a more remarkable immune-inhibiting effect on IFN-γ-treated EPCs when compared with AD-MSCs (n = 5, P<0.05).

MSCs were considered to be immune privileged in previous studies. Our study demonstrated that MSCs could also mildly stimulate allo-PBMCs proliferation. The stimulation ability of UC-MSCs was lower than AD-MSCs, and EPCs had the highest stimulation ability when compared with UC-MSCs and AD-MSCs. When MSCs were mixed with IFN-γ pre-treated or untreated EPCs, MSCs could significantly down-regulate the proliferation of allo-PBMCs. The immuno-modulatory effect of UC-MSCs was greater than AD-MSCs, especially when mixed with IFN-γ pre-treated EPCs (Fig 3).

**Fig 4:**
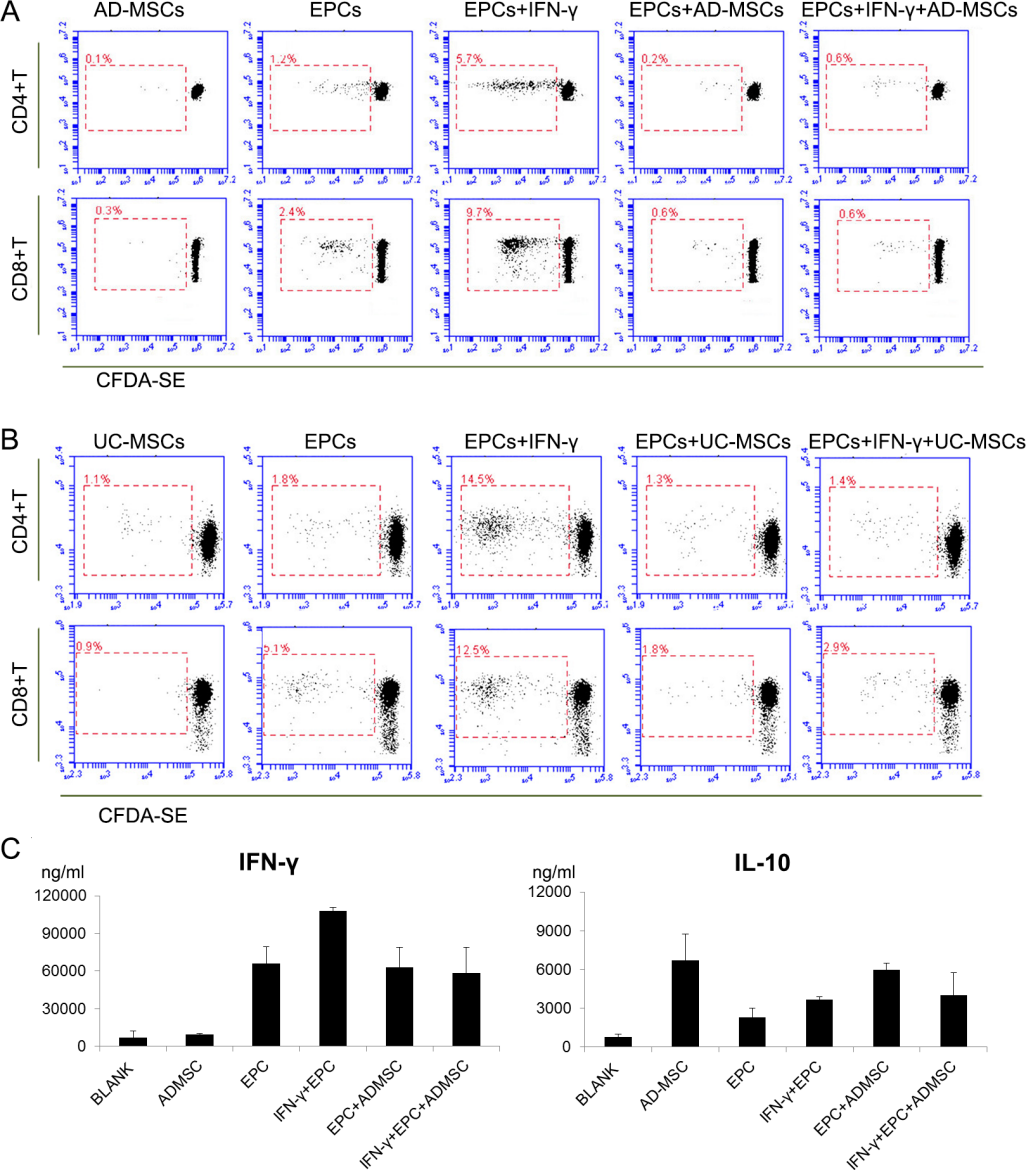
The stimulating effect of EPCs, AD-MSCs and UC-MSCs on the proliferation of T cell subsets and the cytokine secretion of the three kinds of cells when co-cultured with allo-PBMCs. The stimulating effect of EPCs, AD-MSCs and UC-MSCs on the proliferation of T cell subsets was further analyzed. After one week of co-culture, CEDA-SE labeled T cells were stained with anti-CD4 or anti-CD8 antibody and subjected to FACS analysis. A and B, Typical results of 5 independent tests with similar trends. AD-MSCs, UC-MSCs or EPCs alone could hardly stimulate CD4+T or CD8+T proliferation, though the stimulating effect of EPCs was slightly higher. After pre-treatment by IFN-γ, EPCs could strongly stimulate CD4+T and CD8+T proliferation. However, this effect could be significantly inhibited by both AD-MSCs and UC-MSCs (n = 5, P<0.05). C, Cytokine secretion was analyzed after a 7-day co-culture of allo-PBMCs with different stimulators. EPCs can stimulate the production of IFN-γ and IL-10. AD-MSCs can down-regulate the secretion of IFN-γ (EPCs + IFN-γ group vs EPCs + IFN-γ + AD-MSCs group) and up-regulate the secretion of IL-10 (blank vs ADMSCs group, EPCs group vs EPCs + AD-MSCs group) (n = 3, P<0.05).

Furthermore, we analyzed the effect of different stimulator cells on the subsets of T cells by FACS. Though AD-MSCs and UC-MSCs could mildly induce the proliferation of PBMCs, they could not induce CD4+T or CD8+T cell proliferation because of their low immunogenicity. EPCs had a stronger immunogenic effect because they could promote the proliferation of CD4+T and CD8+T cells. This effect was enhanced when EPCs were pre-treated with IFN-γ, and weakened with the addition of AD-MSCs or UC-MSCs (Fig 4).

### EPCs and/or AD-MSCs could affect cytokine secretion of allo-PBMCs

When stimulated by EPCs and/or AD-MSCs, PBMCs secrete various kinds of cytokines. We mainly detected anti-inflammatory cytokine IL-10 and pro-inflammatory IFN-γ in co-culture supernatant. EPCs could induce PBMCs to secrete more IFN-γ than AD-MSCs (P = 0.003). In contrast, AD-MSCs could induce PBMCs to secrete more IL-10 than EPCs (P = 0.01). PBMC secretion of IFN-γ decreased while PBMC secretion of IL-10 increased with co-culture with AD-MSCs (Fig 4).

### UC-MSCs and AD-MSCs can promote vessel formation *in vivo*

**Fig 5:**
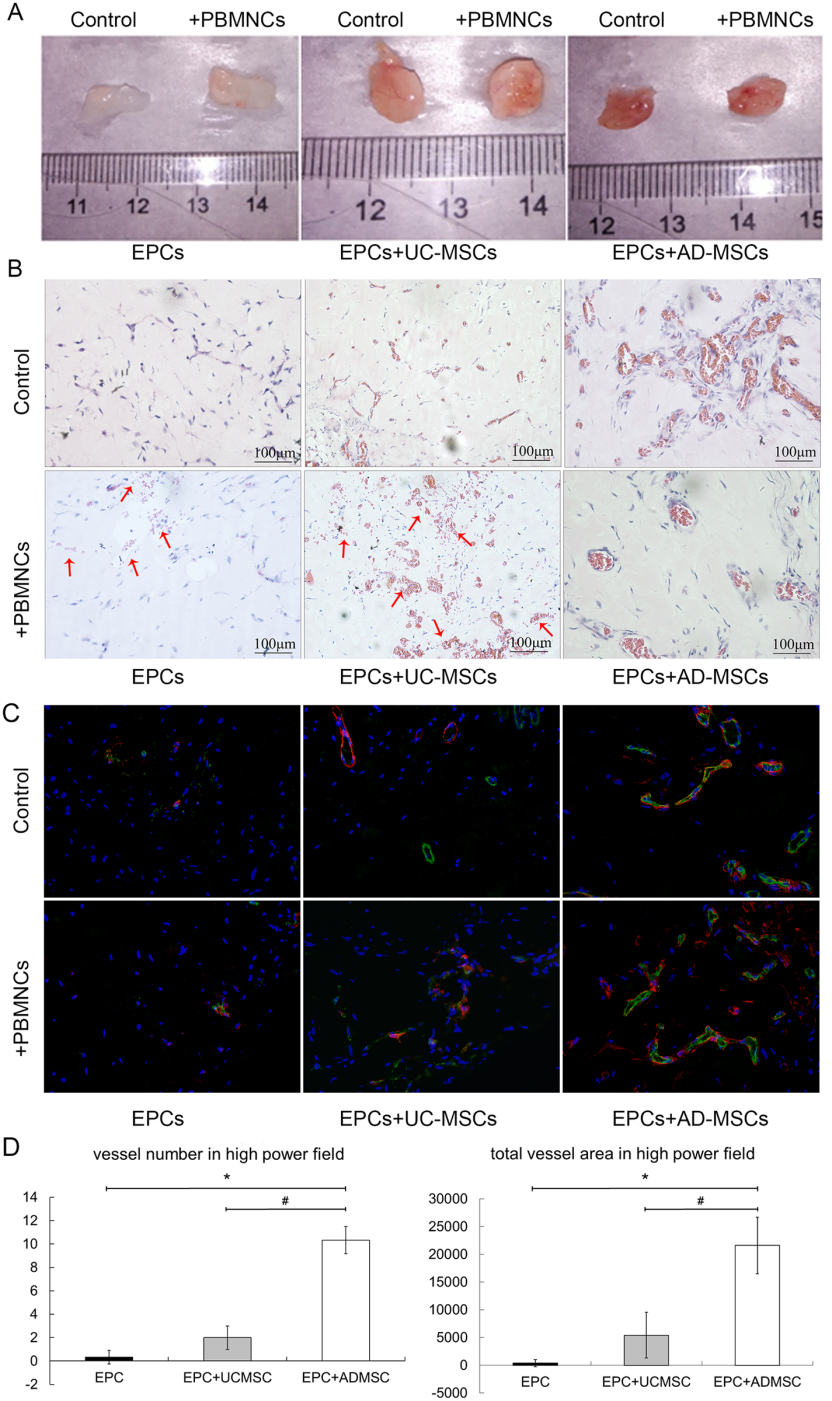
AD-MSCs can promote vessel formation *in vivo*. After EPCs were implanted with/without UC-MSCs or AD-MSCs into SCID mice for 2 weeks, formed vessels in matrigel were subjected to immune-rejection by allo-PBMCs. The results were demonstrated by gross observation of the matrigel grafts (A) or by pathological section observation after H&E staining (B) or immunofluorescence staining (green: CD31; red: SMAα; blue: DAPI. 400×)(C). Implanted cells in matrigel formed vessels that inosculated with host vasculature and were perfused with mouse blood. EPCs implanted with AD-MSCs formed more vessels than EPCs implanted alone or EPCs implanted with UC-MSCs (D). The vessels in EPC+AD-MSC grafts also had significantly larger lumen than other groups (D). After allo-PBMC injection, red blood cell leakage was very obvious in EPC grafts or EPC+UC-MSC grafts (indicated by red arrows). In EPC+AD-MSC grafts, the vessels remained stable, and the red blood cell infiltration could hardly be observed.

Grafts containing AD-MSCs + EPCs (2:3) showed more vessel formation (10.33±1.15 vessels in each high power field) and larger lumen (21599.67±5104.6 total vessel pixel area in each high power field) compared with grafts containing EPCs or UC-MSCs + EPCs (2:3) (0.33±0.58 and 2±1 vessels in each high power field respectively, 381.33±660.49 and 5429.33±4099.05 total vessel pixel area in each high power field)(P<0.01) (Fig 5). After 2 weeks of vessel formation *in vivo*, 2×10^6^ allo-PBMCs were injected into SCID mice, and the results of the immune reaction were compared. We observed obvious red blood cell leakage in grafts containing EPCs or EPCs + UC-MSCs, while no red blood cell leakage was observed in grafts containing EPCs + AD-MSCs (Fig 5).

## Discussion

It is well-known that EPC could be a valuable cellular resource for patients who suffer from cardiovascular disease or ischemic disease [7,16]. However, it is also well-known that EPCs need to be obtained from MHC-matched donors to reduce allo-antigenicity [17]. EPCs are immunogenic, they can activate T cells and they will likely be rejected by the immune system if used for cell therapy and/or tissue engineering [18], which may dramatically affect EPCs’ function and efficiency. The aim of this study was to find a new way to down-regulate the immunogenicity of EPCs and to promote stabilization of the neovascular structure without gene alteration or immune suppressing drugs.

We characterized the *in vitro* and *in vivo* immunogenicity of cord blood EPCs compared to AD-MSCs and UC-MSCs. The EPCs used in our study are CD31+vWF+VEGFR2+CD144+CD105+CD14-CD45-. They are also called late EPCs or endothelial colony forming cells (ECFCs) [19]. In our study, AD-MSCs and EPCs showed a similar expression pattern of MHC-I/II and co-stimulator molecules (CD40, CD80 and CD86) with or without IFN-γ-stimulation. At the same time, UC-MSCs showed weaker immunogenicity when compared with EPCs and AD-MSCs, which was demonstrated by lower expression levels of MHC and co-stimulator molecules in the FACS assay. This difference may be attributed to the discrepancies in the IFN-γ receptor expression.

In the mixed lymphocyte reaction assay, the three types of cells showed slightly different immunogenicity. AD-MSCs acted more like UC-MSCs than EPCs. EPCs had higher levels of immunogenicity, demonstrated by a more powerful ability to stimulate the proliferation of allogeneic PBMCs and CD8+ T cells *in vitro*. UC-MSCs and AD-MSCs could significantly down-regulate the immunogenicity of EPCs, and the immune-regulation effect of UC-MSCs was greater than AD-MSCs. This immune-regulatory effect of MSCs may due to their cytokine secretion pattern [20,21]. In our preliminary experiment, the IL-10 or IFN-γ secretion in EPC or MSC conditioned medium, treated or untreated by exogenous IFN-γ, could not be tested, which means IL-10 or IFN-γ was most likely not secreted from EPCs or MSCs, but from PBMCs. In our study, we found that AD-MSCs could promote the secretion of anti-inflammatory cytokine IL-10 and inhibit the secretion of pro-inflammatory cytokine IFN-γ. Other mechanisms underneath the immune-regulatory effect of MSCs may due to HLA-DR downregulation and indolamin-2,3-dioxygenase (IDO) activation [22].

Our study confirmed the well-reported potential of co-transplantation of EPCs and MSCs to generate more stable functional blood vessels in subcutaneous matrigel plugs [22,23]. The cross-talk between EPCs and MSCs has also been broadly discussed. EPCs can serve as paracrine mediators and regulate the regenerative potential of MSCs via PDGF-BB/PDGFR-β signaling [23]. MSCs can secrete cytokines to promote the survival, proliferation and migration of EPCs, express matrix metalloproteinase to degradate and remodel extracellular matrix. In the present study, we also confirm that MSCs can function as pericytes (SMAα+) and stabilize vascular networks formed by CD31+ endothelial cells. However, difference between the effects of UC-MSCs and AD-MSCs on EPCs has never been compared.

Initial experiments showed that AD-MSCs were more efficient than UC-MSCs in promoting the formation of tubular networks by EPCs both in vitro and in vivo (data not shown). In this study, the benefits of AD-MSCs co-culture in the generation and maintenance of new blood vessels was further confirmed. We found that AD-MSC and EPC co-transplantation promoted a more stabilized neovascular structure in SCID/beige mice. This neovascular structure could even resist immune attack from human allogeneic PBMCs. Although UC-MSCs could also support neovascularization, immune rejection by allo-PBMCs was demonstrated by obvious red blood cell leakage in UC-MSC+EPC matrigel grafts. The distinct advantages of AD-MSCs are due not only to their immune-regulation function but also to their ability to secrete cytokines known to improve capillary tube formation (data not shown). How AD-MSCs regulate EPC immunogenicity is still unclear; however, potential mechanisms may consist of the following: 1. AD-MSCs could positively affect their environment via secreted paracrine factors, including cytokines [21], microRNAs [24] and exosomes [25]. 2. AD-MSCs may affect EPCs by altering their epigenetic markers [26], such as DNA methylation, histone modifications and small non-coding RNA transcripts. 3. AD-MSCs may affect EPC biological function through adhesion molecules such as E-cadherin [27].

Our AD-MSC and EPC co-transplantation model may have some limitations. First, as a model system, matrigel grafts cannot fully mimic a disease condition. We need to further test AD-MSC and EPC co-transplantation through intravenous infusion in a different disease model, such as hind limb ischemia or myocardial infarction. Second, it’s hard to describe the complexity and the whole picture of *in situ* inflammation. For example, we couldn’t find obvious inflammatory infiltration in any of the matrigel plugs after PBMC injection. But we consider it an important first step to analyze immuno-response against implants containing EPCs and MSCs. Third, allo-PBMC injections into SCID mice is not a perfect model analogous to immune rejection in a human recipient. Endothelial cells not only express HLA-I and II antigens but also express other antigens such as polymorphic MICA (MHC class I-related chain A) molecules, which are also antigenic targets for antibody-mediated rejection (AMR) [28]. AD-MSC transplantation alone may not be sufficient to suppress immune reaction caused by EPC transplantation. Fourth, unlike MSC, EPC transplantation is not broadly applied in clinical trials. The safety of EPC transplantation requires further evaluation.

In summary, our results strongly support that AD-MSCs and UC-MSCs can regulate the immunogenicity of EPCs and inhibit the immune rejection caused by EPC transplantation. UC-MSCs are more powerful in immune regulation than AD-MSCs. However, AD-MSCs are more compatible in supporting neovascularization when co-transplanted with EPCs. Therefore, AD-MSCs, especially autologous AD-MSCs, could be used with EPCs to minimize immunological rejection in future vascular disease treatment.
